# Harmonic Vibrational Frequency Simulation of Pharmaceutical Molecules via a Novel Multi-Molecular Fragment Interception Method

**DOI:** 10.3390/molecules28124638

**Published:** 2023-06-08

**Authors:** Linjie Wang, Pengtu Zhang, Yali Geng, Zaisheng Zhu, Shiling Yuan

**Affiliations:** 1 School of Chemical Engineering, Shandong Institute of Petroleum and Chemical Technology, Dongying 257061, China; linjiewang1989@hotmail.com (L.W.); gogogoooliver@163.com (P.Z.); yaligeng0313@163.com (Y.G.); longinces@126.com (Z.Z.); 2 School of Chemistry and Chemical Engineering, Shandong University, Jinan 250199, China

**Keywords:** DFT, molecular modeling, Infrared spectroscopy, multi-molecular fragment interception

## Abstract

By means of a computational method based on Density Functional Theory (DFT), using commercially available software, a novel method for simulating equilibrium geometry harmonic vibrational frequencies is proposed. Finasteride, Lamivudine, and Repaglinide were selected as model molecules to study the adaptability of the new method. Three molecular models, namely the single-molecular, central-molecular, and multi-molecular fragment models, were constructed and calculated by Generalized Gradient Approximations (GGAs) with the PBE functional via the Material Studio 8.0 program. Theoretical vibrational frequencies were assigned and compared to the corresponding experimental data. The results indicated that the traditional single-molecular calculation and scaled spectra with scale factor exhibited the worst similarity for all three pharmaceutical molecules among the three models. Furthermore, the central-molecular model with a configuration closer to the empirical structure resulted in a reduction of mean absolute error (MAE) and root mean squared error (RMSE) in all three pharmaceutics, including the hydrogen-bonded functional groups. However, the improvement in computational accuracy for different drug molecules using the central-molecular model for vibrational frequency calculation was unstable. Whereas, the new multi-molecular fragment interception method showed the best agreement with experimental results, exhibiting MAE and RMSE values of 8.21 cm^−1^ and 18.35 cm^−1^ for Finasteride, 15.95 cm^−1^ and 26.46 cm^−1^ for Lamivudine, and 12.10 cm^−1^ and 25.82 cm^−1^ for Repaglinide. Additionally, this work provides comprehensive vibrational frequency calculations and assignments for Finasteride, Lamivudine, and Repaglinide, which have never been thoroughly investigated in previous research.

## 1. Introduction

Infrared (IR) spectroscopic analysis of biological samples commenced in the 1950s [1], and was speedily put to use in the realm of pharmaceutical research [2]. Infrared spectroscopy records the spectral pattern of samples and expresses the chemical composition as a function of wavenumbers between 400 and 4000 cm^−1^ [3,4]. With advancements in testing technology, Fourier transform infrared (FT–IR) spectroscopy emerged as a potent device for distinguishing and identifying an array of diverse samples, and, indeed, all forms of samples could be characterized thereby. It is a prompt and refined modality for sample characterization whereby the chemical configuration can be analyzed as simple molecules, revealing specific absorption bands in the FT–IR spectra [5,6].

The vibrational frequencies serve as a distinctive signature for chemical compounds, and are commonly employed in the identification and characterization of organic and inorganic specimens [7,8,9]. The infrared spectrum is derived from the vibrational motion of the molecules, and has significant implications in the qualitative and quantitative exploration of matter [10]. At present, infrared spectroscopy is a widely used and easily accessible characterization tool that plays a vital role in the understanding and utilization of materials [11]. Although advanced techniques, such as nuclear magnetic resonance and mass spectrometry, offer high-resolution molecular analysis, their prohibitive costs limit their accessibility. Therefore, obtaining material property information using simple and cost-effective methods is particularly meaningful for many research institutions and manufacturers. However, empirical guidance still dominates our interpretation of infrared spectra, presenting significant challenges in analyzing weak functional groups and fingerprint regions. Furthermore, infrared spectrometers have natural limitations, including peak overlapping caused by the proximity of strong vibration peaks, which impedes spectral analysis [12]. Therefore, it is urgent to develop a new perspective to address these problems effectively.

In recent years, the application of DFT in the field of pharmacy has experienced a rapid progression [13,14,15]. Quantum chemistry has played an important role in the field of drug research and development, and a large number of investigations have been conducted by means of computer-simulated infrared spectroscopy [16,17,18]. The force fields in these calculations provide potential energy surfaces, which map out the relative energy of a configuration of atoms in a molecule. One common type of force field is DFT-based, which includes the popular GGA [19]. The PBE exchange–correlation functional is a widely used form of GGA and has been shown to accurately predict and represent vibrational spectra [20,21,22]. In our work, we focus on the utilization of the GGA/PBE level to simulate vibrational spectra accurately.

However, up to the present, all of the research works have been optimized and frequency-calculated by constructing single-molecular structures with different functionals and basis sets [23,24]. The molecules were placed in a vacuum environment, and no heed was given to the spatial configuration and hydrogen bonding of the molecules. As a result, the outcomes were frequently imprecise, notably for molecules with intermolecular hydrogen bonds. We considered the reasons for this and found that the input single molecule models might differ significantly from the actual molecular state, which can be attributed to two factors. First, the first step of all calculations is model optimization, which is typically performed using the single-molecular model, neglecting the spatial hindrance caused by the crystal or amorphous state. This can lead to changes in molecular structure, which significantly affect vibrational frequencies and result in errors. Second, materials with functional groups in crystals or amorphous states are often connected through intermolecular hydrogen bonds (Figure S1), which have significant impacts on infrared spectral peaks. However, when performing frequency calculations with the single-molecular model as input, the molecular hydrogen-bond, or conjugation, information is lost, leading to increased computational errors. Therefore, when compared with experimental frequencies, theoretically-calculated harmonic frequencies were commonly discovered to be 10–15% excessively high, partly due to the exclusion of anharmonic effects and the incomplete incorporation of electron correlation [25]. To solve this problem, scale factors of different functionals were proposed [26,27,28].

The development of scale factor calculation has been divided into two stages. In the first stage, the factors were determined as an average of the experimental/theoretical ratios for individual modes utilizing numerous molecules. For instance, Hout, Levi, and Hehre (1982) [29] determined the scale factor of MP2/B3LYP (0.921, inverse of 1.086) using 36 molecules via this method. Secondly, the subsequent development of analytic second derivatives with finite difference techniques was proposed to increase the availability of theoretical frequencies. This method was validated by Simandiras, Handy and Amos [30], indicating higher accuracy. Other methods, such as employing the E(ZPE) equation, have also been proposed to calculate scale factors [25]. However, the methods and parameters in the equations provide empirical and statistical results from the modification of a large number of small molecular groups, which may not be applicable for molecules with distinct spatial configurations and intermolecular interactions. Furthermore, confusingly, different literature has shown different values of scale factor for the same DFT. For example, for HF/6-31G(d), Qi (2000) [31], Irikura (2005) [32] and Barone (2004) [33] demonstrated values of 0.9590, 0.8982 and 0.8929, respectively. Therefore, a new simulation method must be proposed to improve the calculation’s accuracy and eliminate the perplexity of scale factor selection.

Harmonic approximation assumes that vibrations are harmonic and linear, which simplifies calculations but has severe limitations when it comes to describing real-world vibrational phenomena [34]. The harmonic model assumes that the potential energy function for a molecule is quadratic and that each vibrational mode oscillates with a single frequency. This assumption holds when the vibrations are sufficiently small that the restoring force in the bond potential can be approximated as linear, which makes it a reasonable starting point for predicting vibrational spectra. However, this approach breaks down when larger amplitude vibrations occur. Large-amplitude vibrations result in the stretching or bending of bonds beyond their equilibrium positions, which leads to the appearance of higher-order effects, including anharmonicity [35]. Anharmonicity describes the deviation of vibrational modes from harmonic behavior, occurring when interactions between different vibrational modes become significant, causing nonlinear coupling between the modes. This coupling can result in large deviations from harmonic behavior, altering vibrational frequencies and intensities [36]. In addition to anharmonicity, environmental factors can significantly affect the behavior of molecules in ways that the harmonic model does not account for. However, anharmonic analyses are computationally expensive and still not applicable to all types of molecules. Additionally, although some more sophisticated methods, such as B3LYP, M06-2X, and *ω*-B97X-D, can better describe anharmonic effects, the accuracy of the results is limited by various drawbacks of the single-molecular model. While the GGA/PBE method we use does not directly consider anharmonic effects in predicting vibrational frequencies, it is still a very common computational method in this field at present [20,21,22]. Therefore, in this paper, a new idea to construct a multi-molecular model containing molecular environment information and intermolecular conjugation information under harmonic approximation, so as to improve the accuracy of vibrational spectrum calculation and save computational resources, is proposed.

There are several reasons why including multiple molecules in our calculations can help reduce the limitations of the harmonic approximation and improve accuracy. Firstly, a multi-molecular model can take into account intermolecular interactions, which are crucial in many chemical processes, such as hydrogen bonding or protein–ligand binding. Neglecting such interactions can lead to significant errors in spectroscopic predictions. Secondly, in actual multi-molecular systems, the interactions between different molecules can change the frequency and intensity of vibration modes. For example, in a multi-molecular hydrogen-bond model, hydrogen bonding typically leads to a significant enhancement of anharmonic effects in the vibration modes and introduces higher-order vibration terms [37]. When we use a single-molecular model, we may neglect the influence of non-covalent interactions in multi-molecular systems and treat molecular vibrations as purely internal vibrations, which is a harmonic approximation and may lead to significant discrepancies with the experimental results. On the other hand, using a molecular model containing multiple molecules can better consider the non-covalent interactions and environmental effects between molecules, and, thus, more accurately describe the vibrational behavior and spectral characteristics of molecules. Additionally, the inclusion of multiple molecules in the model can lead to a more realistic representation of the sample material analyzed by spectroscopic techniques. We believe that including assets such as multiple molecules within the limitations of the harmonic approximation provides a useful and computationally efficient tool for analyzing vibrational spectra of light-to-medium-sized molecules.

In this paper, a novel vibrational simulation method was developed to facilitate the correct assignment of infrared spectra. The pharmaceutical molecules Finasteride (FIN), Lamivudine (LAM), and Repaglinide (REP) were employed as models for this purpose. The theoretical and experimental results of the vibrational harmonic spectra were compared to validate the new method. Three models were constructed in the Material Studio program, and vibrational simulations of single molecules, multi-molecular fragments, and central molecules were conducted and compared with the scaled spectrum.

## 2. Results and Discussion

### 2.1. Traditional Single-Molecular Vibration Analysis

**Figure 1:**
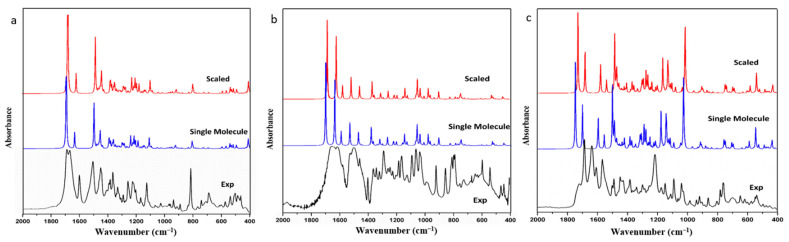
The infrared spectra of experimental, simulated single molecule, and simulated single molecule after scaling for (**a**) FIN, (**b**) LAM, and (**c**) REP.

Frequency simulations conducted thus far have only involved single molecules and scaling with scale factors. However, calculated spectra with scale factors still exhibit significant deviation from experimental results, and the scale effect varies across different molecules and functional groups, even with the same factor. To verify this problem, we extracted FIN, LAM, and REP single molecules as model molecules, optimized and calculated their harmonic vibration frequencies and infrared spectra, and compared these with the experimental spectra. The infrared spectra for the experimental spectra, the simulated single molecule, and the simulated single molecule after scaling for FIN, LAM, and REP are displayed in Figure 1. A portion of observed and calculated vibrational frequencies, along with their respective dominant normal modes, for FIN, LAM, and REP are listed in Table 1. The scaled wavenumbers are presented after being scaled by a scaling factor of 0.99 [38]. All assignments are provided in the Supplementary Materials (Tables S1–S3).

As depicted in Figure 1, the calculated and scaled infrared spectra of single molecules for FIN, LAM, and REP were similar to the experimental results. The simulated and experimental spectra exhibited similar peak shapes at corresponding wavenumbers, indicating high calculation accuracy. MAE and RMSE were used in the linear regression analysis to assess the deviations between the theoretical and experimental structural parameters. The MAE and RMSE between the calculated, scaled, and experimental infrared spectra for each molecule were compared, yielding values of 12.99 cm^−1^ and 29.80 cm^−1^, 52.17 cm^−1^ and 109.50 cm^−1^, 26.48 cm^−1^ and 57.51 cm^−1^ for the single-molecular simulation; and 16.99 cm^−1^ and 27.14 cm^−1^, 50.56 cm^−1^ and 101.75 cm^−1^, 29.39 cm^−1^ and 44.34 cm^−1^ for the scaled spectra of FIN, LAM, and REP, respectively. Therefore, after scaling, the infrared spectrum of LAM exhibited greater similarity to the experimental data, while the opposite was observed for FIN and REP. Thus, for macro-molecules, particularly those with complex configurations, the scale factor may not be entirely applicable. Nonetheless, at hydrogen-bonding sites, the scaled vibrations outperformed the single-molecular simulations. In the case of FIN (Table 1), the N-H and C=O stretching were calculated as 3517.12 cm^−1^, 3514.26 cm^−1^, 1695.16 cm^−1^, 1693.63 cm^−1^ for the single-molecular simulation and 3481.95 cm^−1^, 3479.12 cm^−1^, 1678.21 cm^−1^, 1676.69 cm^−1^ for the scaled spectra, respectively. In these bands, the scaled vibrations exhibited better fit to the experimental spectra, which was consistent with the behaviors observed in LAM and REP (Table 1). This result correlates with a slight reduction in the value of the RMSE after scaling. Consequently, the scale factor was primarily employed to adjust the vibrations of hydrogen-bonded functional groups, and had minimal impact on the overall molecule, particularly in the skeleton vibration and fingerprint region.

This could be due to the fact that the optimization was searching for the smallest potential point by changing the atomic situation around the input structure. However, most single-molecular models are derived from crystal lattices, which simplifies the structural optimization process, but results in a loss of information regarding intermolecular interactions and spatial arrangements. Consequently, this leads to inaccuracies in calculation and vibration assignment. Although the use of a scale factor improves the similarity between the calculated and experimental results to some extent, it is an empirical and statistical value that is insensitive to intermolecular interactions and spatial configurations. This shortcoming may lead to unsatisfactory outcomes. Therefore, it can be concluded that the traditional single-molecular simulation method, even with the inclusion of a scale factor, is not a reliable approach for accurately calculating molecular frequencies.

### 2.2. Central-Molecular Simulation Analysis

**Figure 2:**
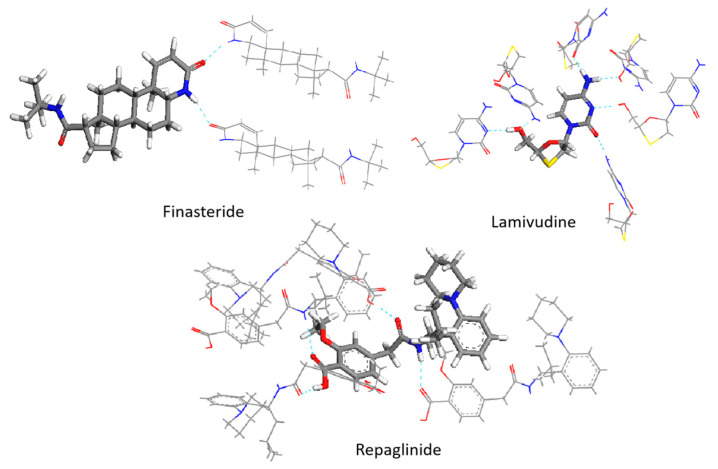
The minimum multi-molecular repeating units of FIN, LAM, and REP (Hydrogen bonds are illustrated by blue dashed lines). N atoms are colored blue, O atoms are colored red, H atoms are colored white, C atoms are shown in gray, and S atoms are shown in yellow.

**Figure 3:**
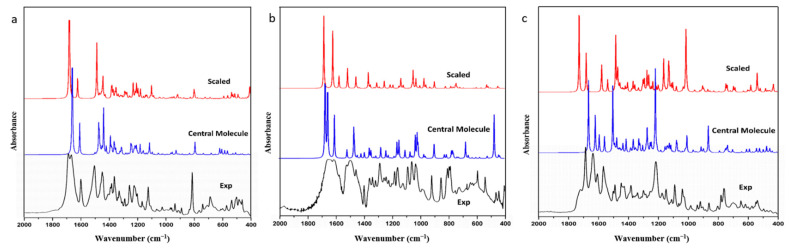
The infrared spectra of experimental, simulated central-molecular and simulated single-molecular models after scaling for (**a**) FIN, (**b**) LAM, and (**c**) REP.

In order to investigate the impact of spatial configuration on vibrational simulation, we constructed the minimum repeating space structure unit to preserve the configuration as much as possible while minimizing computational resources. To accurately depict the true configuration and intermolecular interactions, the crystal cells of FIN, LAM, and REP were imported in the MS program and supercells of 2 × 1 × 1, 3 × 3 × 1, 2 × 1 × 3 were constructed, respectively. The structures of supercells are shown in Figure S1. We extracted the minimum repeating unit from the supercell of molecules and optimized it using the GGA/PBE functional. After optimization, the central single molecule was then extracted and calculated the frequencies at the same level of theory. The minimum multi-molecular repeating units for FIN, LAM, and REP are shown in Figure 2. The scaled, central-molecular calculated and experimental spectra for the three drugs are also shown in Figure 3. A portion of available experimental and theoretical vibrational frequencies with their respective dominant normal modes for FIN, REP, and LAM are listed in Table 2. All the assignments are included in the Supplemental Materials (Tables S1–S3).

From Figure 3, it is apparent that the spectra of the central molecule were superiorly compatible than those of the single molecule and the scaled spectrum. This observation strongly implies that spatial configuration played a pivotal role in the frequency calculations of all three molecules. Furthermore, the MAEs and RMSEs between the experimental infrared spectra and the simulated and scaled spectra of the central molecule for FIN, LAM, and REP were compared. The results showed that the MAE and RMSE for the central-molecular model were 9.34 cm^−1^ and 21.81 cm^−1^, 20.36 cm^−1^ and 30.40 cm^−1^, 23.12 cm^−1^ and 52.21 cm^−1^ for FIN, LAM, and REP, respectively, whereas the corresponding values for the scaled spectra were 16.99 cm^−1^ and 27.14 cm^−1^, 50.56 cm^−1^ and 101.75 cm^−1^, 29.39 cm^−1^ and 44.34 cm^−1^, as listed in Table 2. The significant reduction observed in the RMSE of LAM was primarily due to the precise calculation of the infrared vibration at the hydrogen-bonding site (*ν_as_*N_3_H_3–4_) by the central-molecular model. Therefore, the central-molecular model for vibrational simulation was found to be more accurate than the single-molecular and scaled results for all three pharmaceutics. Moreover, for the hydrogen-bonded functional groups, the central-molecular model was also found to yield better fits to the experimental spectra.

**Figure 4:**
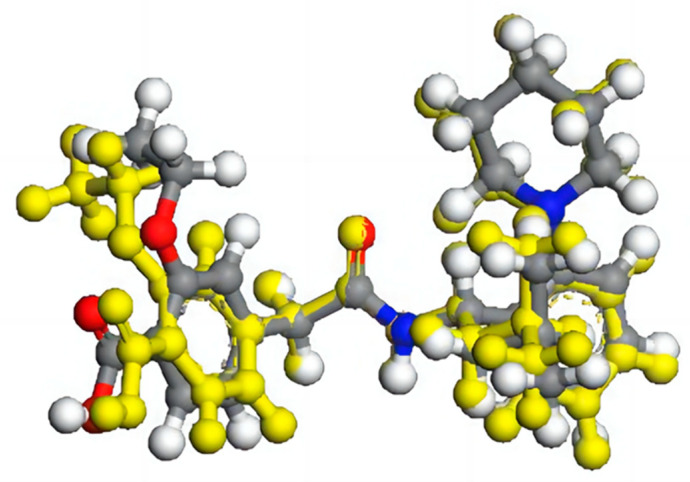
The structural comparison of REP molecule after optimization with single-molecular and multi-molecular repeating units. The multi-molecular optimized structure is colored yellow.

After optimization, the geometrical parameters (bond length, bond angle, and dihedral angle) of the single molecule may differ from those of the central molecule due to the lack of intermolecular interactions in the former case. For instance, in the case of the REP molecule, the ethoxy and carboxyl groups were twisted by 4.06 and 6.22 degrees, respectively, after optimization by the single-molecular and multi-molecular repeating units (as observed in Figure 4). Therefore, multi-molecular units can retain the configuration when optimized with functional theory, leading to more accurate calculations of frequencies, which are closer to experimental values.

One may be inclined to assume that the single molecule extracted from the crystal cell has the same configuration as the real molecule and performs optimally. However, it was observed that the calculated spectra of the single molecule without optimization differed greatly from the experimental results, and even displayed imaginary frequencies. This could be attributed to the fact that frequency simulations in Gaussian are based on the first derivative of the potential function being zero. As such, structures without optimization cannot be processed using the Schrodinger equation.

Therefore, when compared to the single-molecular model and scaled spectra, the central-molecular model that was optimized using multi-molecular repeating units exhibited a better fit to the experimental spectrum. However, the central-molecular model only provided configuration information and lacked information on intermolecular interactions. As a result, the calculated frequencies at the hydrogen-bonding donor and receptor sites still deviated significantly from the experimental results. For instance, for LAM (Table 2), the experimental value of N_3_-H_3_ stretching was 3383 cm^−1^, which was calculated as 3620.42 cm^−1^ for scaled spectra and 3484.85 cm^−1^ for the central-molecular calculation, respectively. For REP (Table 2), the experimental value of O_4_H_36_ peak was at 3428 cm^−1^, which corresponded to 3162.17 cm^−1^ and 3566.16 cm^−1^ for the central-molecular and scaled spectra, respectively. In general, the accuracy of the central-molecular model was similar to that of the scale factor method. To overcome this limitation, a new multi-molecular fragment interception method was proposed.

### 2.3. Multi-Molecular Fragment Interception Simulation Analysis

The discussion of the central-molecular model analyzed the influence of configuration on vibrational simulation. However, the calculated frequencies of hydrogen-bonded functional groups exhibited significant deviation from experimental results. To solve this problem, a multi-molecular fragment interception method was proposed. This model was derived from the optimized multi-molecular repeating unit by eliminating long-distance molecular fragments with low conjugation and charge effects around the central molecule. To accurately depict the true configuration and intermolecular interactions, the crystal cells of FIN, LAM, and REP were imported into the MS program and supercells of 2 × 1 × 1, 3 × 3 × 1, 2 × 1 × 3 were constructed, respectively (Figure S1).

**Figure 5:**
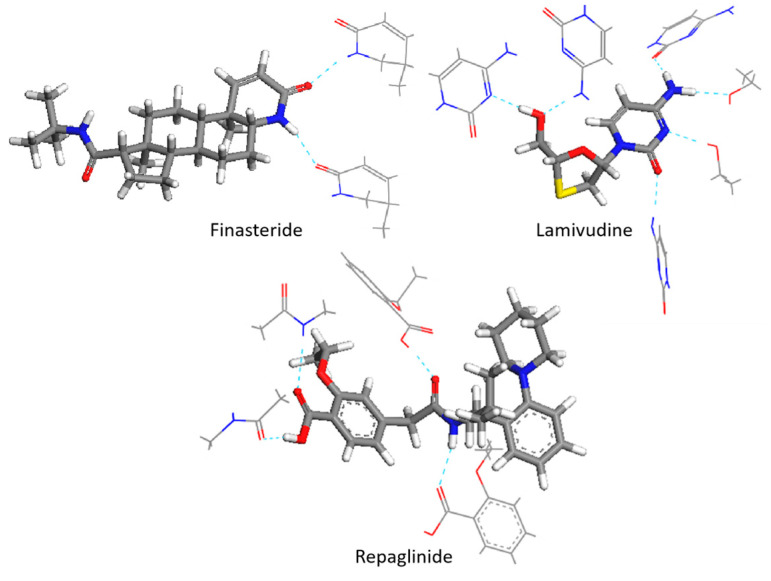
The multi-molecular fragment interception models of the three drugs.

**Figure 6:**
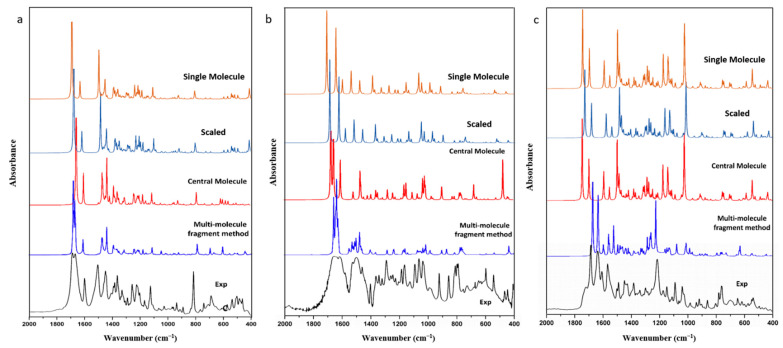
The infrared spectrum of experimental, simulated multi-molecular fragment, simulated central molecule, simulated single molecule and scaled single molecule for (**a**) FIN, (**b**) LAM, and (**c**) REP.

To retain the space configuration and hydrogen-bond information as far as possible, we optimized the repeating unit using the GGA/PBE functional, rather than a single molecule. Additionally, to reserve the computing resources, the long-distance molecular fragments with low conjugation and charge effect around the central molecule were intercepted to construct the multi-molecular fragment (see Figure 5). The frequency of the molecules was then calculated using the same functional, and the infrared spectrum cooperation are shown in Figure 6. Upon comparison of the calculated spectra with the experimental spectra, numerous matching bands in this region were identified and are presented in Table 3, for FIN, LAM, and REP.

The calculated spectra of the multi-molecular fragment exhibited greater similarity to the experimental spectra in terms of frequencies and peak shapes for all three drug molecules (see Figure 6). This phenomenon suggests that the multi-molecular fragment model, which includes the original space configuration and intermolecular interactions, is superior to traditional scaled single-molecular calculations for frequency simulation. Both MAE and RMSE were calculated to evaluate the performance of the central-molecular, single-molecular, and multi-molecular fragment models. The MAE values for the central-molecular, single-molecular, and multi-molecular fragment models were 9.34 cm^−1^, 20.36 cm^−1^, and 23.12 cm^−1^ for FIN, and 8.21 cm^−1^, 15.95 cm^−1^, and 12.10 cm^−1^ for the multi-molecular fragment model. In addition, the RMSE values were also calculated and compared with the MAE values. The RMSE values for FIN were 29.80 cm^−1^, 21.81 cm^−1^, and 18.35 cm^−1^ for the single-molecular, central-molecular, and multi-molecular fragment models, respectively. For LAM, the RMSE values were 109.50 cm^−1^, 30.40 cm^−1^, and 26.46 cm^−1^, and for REP, the RMSE values were 57.51 cm^−1^, 52.21 cm^−1^, and 25.82 cm^−1^. Therefore, the utilization of the multi-molecular fragment model resulted in a considerable reduction in the MAE and RMSE values. These results indicate that the multi-molecular fragment interception model outperformed both the single-molecular and central-molecular models in terms of ability to accurately predict vibrational frequencies. Therefore, the multi-molecular fragment interception model for vibrational simulation was more accurate than the other models described above for all three pharmaceutics. Additionally, the frequencies calculation with the multi-molecular fragment model for hydrogen-bonded functional groups also demonstrated the best performance relative to empirical spectra. For instance, for FIN in Table 3, the C_3_-O_1_ and C_19_=O_2_ stretching vibrations were calculated to be 1664.17 cm^−1^ and 1662.83 cm^−1^ for the central-molecular model, and 1681.01 cm^−1^ and 1665.21 cm^−1^ for the multi-molecular fragment model, respectively. The experimental frequencies for these two vibrations were 1688 cm^−1^ and 1668 cm^−1^, indicating that the multi-molecular fragment interception model was a better fit to the experimental spectrum. Similar conclusions could be drawn for the REP and LAM molecules.

The reason for the improved accuracy of the multi-molecular fragment model may be attributed to the fact that the model was obtained by intercepting a fragment from the optimized minimum multi-molecular repeating units of hydrogen bonds, which retained both the hydrogen-bonded information and the original configuration. Compared to the central-molecular model, the multi-molecular fragment model removed the long-distance molecular fragments with low conjugation and charge effect around the central molecule, and, thereby still retaining the hydrogen-bonded information as much as possible. Moreover, during the optimization and interception processes, the central molecule of the multi-molecular fragment model was able to maintain its configuration nature and intermolecular interactions, unlike a single molecule in vacuum. Consequently, using a single-molecular model simplified the atomic positions, velocities, and potential energy and yielded a considerable discrepancy between the calculated results and the actual situations. Additionally, a single-molecular model is incapable of describing intermolecular interactions and environmental factors, such as steric hindrance and lattice constraints. In contrast, a multi-molecular model addresses intermolecular interactions between neighboring molecules, encompasses hydrogen bonding, electrostatic, and van der Waals forces, and calculates infrared resonance for complex systems. In addition, the multi-molecular model also considers the interactions between the molecule and its surrounding environment. Compared to the single-molecular model, the multi-molecular model provides better characterization of the molecular spatial structure, and functional group structure, and offers a realistic portrayal of macroscopic substance states, enhancing computational accuracy. The multi-molecular model also encompasses intramolecular resonance effects, dense packing effects, and nonlinear effects, enabling a more comprehensive depiction of molecular vibration and physical–chemical processes, thereby improving the prediction of vibrational frequencies.

As a result, the new method of multi-molecular fragment interception effectively overcomes the limitations of traditional single-molecular vibrational simulation and outperforms other methods with the smallest simulation deviation. It essentially preserves the configuration information and intermolecular interactions, and significantly improves the calculation accuracy, offering a more critical and time-efficient approach for frequency simulation.

### 2.4. Absolute Error Analysis

**Figure 7:**
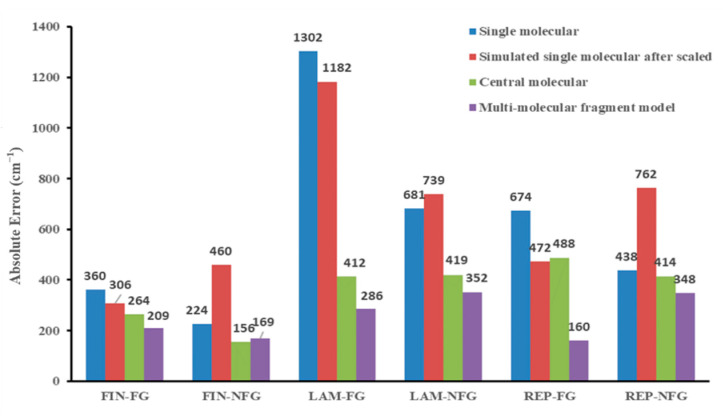
Absolute errors in functional group (FG) and non-functional group (NFG) vibrations of FIN, LAM, and REP.

To investigate the sources of absolute errors (AEs) among different models, we classified the vibrational frequencies of three types of drug molecules into functional group vibrations and non-functional group vibrations. We then studied in detail the changes in AEs caused by different models, as shown in Figure 7.

The results showed that for functional group vibrations, the scale factor had some accuracy improvement effect on the correction of the single-molecular model results, but the effect was generally poor and fluctuated greatly. In the non-functional group set, the scale factor correction increased the calculation error, instead of lowering it. Therefore, the mechanism of adding scale factors sacrificed the calculation accuracy of skeletal vibrations to promote a higher fit between functional group vibrations and experimental values, although this increase in accuracy was not significant (see in Figure 7).

In addition, for functional group vibrations, although the center-molecular model comes from the optimized structure of multi-molecular models, it does not contain intermolecular hydrogen-bonding, or conjugated, information, but the center molecule model has a significantly reduced error compared to the single molecule model. This may be due to two reasons. First, the center molecule model inherits the advantages of the multi-molecule model, and the averaged molecular structure composed of multiple molecules is more similar to the actual molecular structure. Therefore, by averaging multiple molecules and taking the center molecule, the compression or deformation of the actual molecular structure caused by strong interaction forces such as hydrogen bonding can be eliminated. Secondly, considering the intermolecular interactions together with the individual atomic structure in the optimization process can improve the accuracy of calculating the frequency of hydrogen-bonding functional groups in the center molecule model. However, the center molecules of REP did not show small relative AE values, and the center molecule models of FIN and LAM molecules showed great differences in accuracy improvement. This indicates that there is still considerable instability in using center molecule models for vibrational frequency calculation. For the non-functional group region, the analysis of the AEs between the center molecule and the multi-molecule fragment model shows that the AEs of the two models are very similar, and the decrease in AEs compared to the single molecule model is also similar. This indicates that the improvement in calculation accuracy of the non-functional group region is mainly due to the fact that the molecular structure is closer to the true state after optimizing the overall molecular structure of the multi-molecule repeating unit, and the effect of hydrogen bonding information on the vibrational calculation optimization of the non-functional group region is not significant. This conclusion is also easy to understand because there is no hydrogen-bond generation in the non-functional group region, so the addition of multi-molecule hydrogen bonds has a very small effect on vibrational calculation.

Additionally, we can also illustrate that for FIN, LAM, and REP, the reduction in MAE values of the multi-molecular fragment model and single-molecular scale model were 8.07 cm^−1^, 8.96 cm^−1^ and 57.04 cm^−1^, 18.94 cm^−1^ and 28.36 cm^−1^, 13.36 cm^−1^ in the functional group and non-functional group regions, respectively. For LAM and REP, the improvement of computational accuracy in the functional group regions had a more significant effect on reducing the overall MAE, whereas for FIN, the contribution of both functional group and non-functional group vibrations to the overall error was similar. Interestingly, for all three drug molecules, the magnitude of MAE reduction in the multi-molecular fragment model in the functional group region (LAM > REP > FIN) was consistent with the variational trend of intermolecular hydrogen bonds or interactions. This indicates that, for multi-molecular models, the more intermolecular hydrogen bonds present, the greater the reduction in errors observed in functional group vibrations. This may be due to the retention of hydrogen bonding and conjugation information in the multi-molecular model, leading to higher computational accuracy at the hydrogen-bonding connection sites during vibrational frequency calculation.

In conclusion, the improvement in accuracy of the center-molecular and multi-molecular fragment models in the non-functional group region was mainly due to the molecular structure being closer to the true state, and the molecular conjugation information having no significant effect on the accuracy improvement of this part. In the functional group region, both molecular structure and intermolecular conjugation information had significant impacts on the calculation accuracy of vibrational frequencies. The more hydrogen bonding among molecules in the original structure, the greater the contribution of intermolecular conjugational systems to the reduction of MAE. However, the center-molecular model did not have good accuracy performance in all drug molecule models, while the multi-molecular fragment model showed the lowest error values among all models for all drug molecules.

### 2.5. Computational Time Comparison

To provide a quantitative comparison of the computational performance among different models, we conducted structural optimization and vibrational frequency calculations for all models using the same computer and computational resources. The time required for each calculation is summarized in Table 4.

Traditional single-molecular models have the smallest number of atoms, and requires the least amount of time for configuration optimization and vibrational frequency calculation, with a total calculation time not exceeding 3 h. In our paper, the central-molecular and multi-molecular fragment models both used minimal molecular repeat units with hydrogen bonds as initial structures for configuration optimization, causing a significant increase in the time required for this step. Compared to the single-molecular model, FIN, LAM, and REP required an additional 3.91 h, 15.30 h, and 27.95 h, respectively. In the vibrational frequency calculation stage, the central-molecular model took a similar amount of time as the single-molecular model because only one molecular unit was involved. Whereas, due to the inclusion of a large number of hydrogen bonds and conjugated structural fragments in the multi-molecular fragment model, its frequency calculation took approximately 2 h longer than that of the single-molecular model.

However, when selecting drug molecules as calculation models to demonstrate the applicability of new methods more convincingly, we specially selected drug molecules with a high number of atoms, complex structures, and representative hydrogen-bond numbers. For small drug molecules, commonly used for vibrational analysis verification, such as aspirin, the calculation time using the multi-molecular fragment model was only 0.29 h. Moreover, we were pleased to find that although the overall calculation time was longer due to various reasons, including the lack of professional computing servers, our calculations could still be completed within roughly one day. Achieving high computational accuracy is critical in ensuring precise spectral analysis, as even small infrared spectral peak shifts can cause significant analytical errors. Thus, considering the significant reduction in MAEs and the instability observed in the prediction results of the central-molecular model (Figure 7), we regarded the additional time cost as being justified.

The key findings from our study also have several important implications for molecular simulation and drug design. Firstly, the improved accuracy of the multi-molecular fragment interception model suggests that accurately describing intermolecular interactions is critical for accurate vibrational frequency calculations. This finding highlights the importance of advanced modeling approaches that accurately capture the complex interplay between molecules in biological systems. Secondly, this method can be applied to a wide range of molecular systems and provide insights into their structural and functional properties. By accurately calculating vibrational frequencies, the new approach can aid in the interpretation of IR spectra and elucidation of molecular structure and function. This capability is particularly relevant in drug design, where understanding the vibrational properties of drug molecules is critical for predicting their behaviors in vivo. Additionally, this method provides highly accurate guidance for the determination of supramolecular structures, such as co-crystals or co-amorphous systems, enabling precise structural characterization. Thirdly, it has potential applications in optimizing drug formulations and identifying potential drug candidates with desired properties. By accurately describing the intermolecular interactions that contribute to the stability and efficacy of drug formulations, the new approach can aid in the development of more effective and efficient drug delivery systems. Additionally, by accurately describing the vibrational modes associated with specific functional groups, this method can facilitate the identification of potential drug candidates with specific properties or functional groups. We believe that this approach has significant implications for molecular simulation and drug design, and has the potential to contribute to the development of more effective and efficient drug design and development strategies.

## 3. Materials and Methods

### 3.1. Materials

Finasteride, Repaglinide, and Lamivudine were obtained from Hunan Qianjin Xiangjiang Pharm. Inc. (Zhuzhou, China). Ethanol was obtained from Sinopharm Chemical Reagent Co., Ltd. (Shanghai, China). The chemicals were used as received from the companies without further purifications.

### 3.2. Preparation of Drug Crystalline Forms

The Finasteride form I [39] and Repaglinide form I [40] were obtained by recrystallization from absolute ethanol by means of the slow evaporation method and ethanol/water (2:1) by the solvent–antisolvent method, respectively. The received samples were dried under vacuum for 48 h at 313 K.

The Lamivudine form II [41] was produced by heating a suspension of Lamivudine in industrial methylated spirit to reflux to obtain a clear solution. The solution was filtered while hot and half the amount of the solvent from the filtrate was distilled. Then, heating was stopped and the concentrated solution was seeded with authentic form II crystals. The seeded solution was then cooled from 353 K to 298 K for one hour. After further cooling of the the suspension to 288 K and stirring it for an hour, it was filtered and washed with IMS and then dried to give the Form II crystals.

The precipitates were stored in a desiccator until use in the experiment.

### 3.3. FT–IR

FT–IR patterns were recorded using a NICOLET 380 FT-IR spectrometer (Townsend, MA, USA) at wavelengths of 4000–400 cm^−1^. Samples were prepared in KBr pellets by grinding Ca. 1 mg of the drug with KBr and the resolution was 2 cm^−1^.

### 3.4. Computer Details

**Figure 8:**
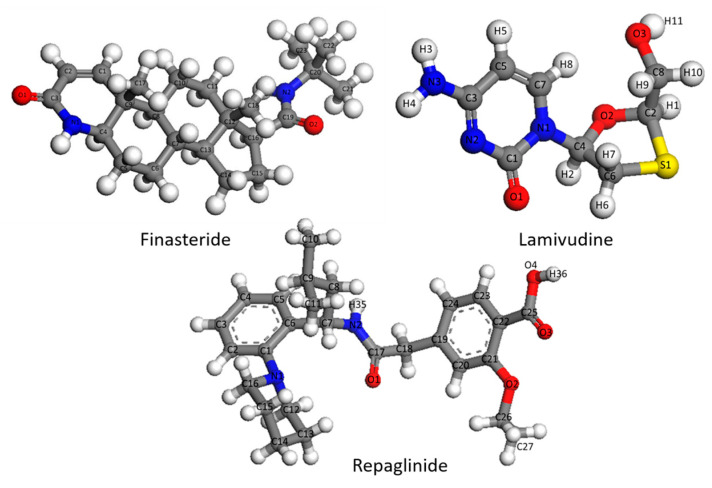
The spatial configuration and atom numbering schemes of Finasteride, Lamivudine, and Repaglinide.

The spatial configuration and atom numbering schemes of Finasteride, Repaglinide and Lamivudine molecules are given in Figure 8. Initial crystalline cells of Finasteride (form I, CCDC Code: WOLXOK02), Repaglinide (form I, CCDC Code: JOHKUM) and Lamivudine (form II, CCDC Code: RUKHAG) were obtained from the Cambridge Crystallographic Database (CCDC, Cambridge, UK) with Conquest 1.8 software (CSD, version 5.27, November 2006 plus 31 updates, Conquest version 1.8).

**Figure 9:**
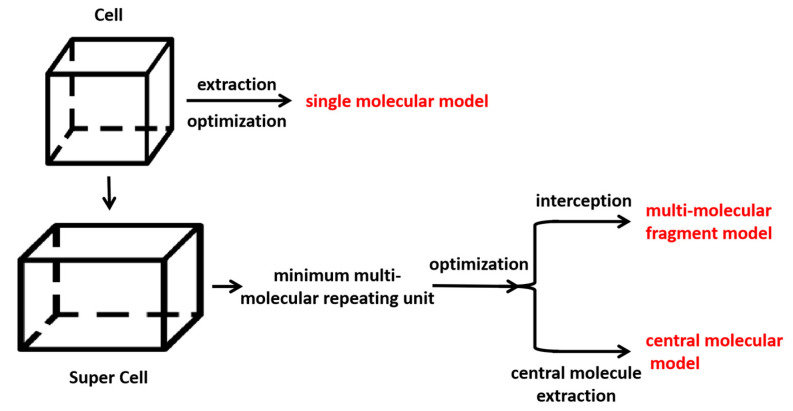
The construction process of the three models.

For each pharmaceutical molecule, we created three distinct models: the single-molecular model, the central-molecular model, and the multi-molecular fragment model, with the single-molecular model serving as a control group. The single-molecular model was a single molecular structure obtained by optimizing the molecular structure extracted from the crystal cell. The central-molecular and multi-molecular fragment models were optimized with multi-molecular hydrogen-bonded repeating units. The minimum multi-molecular hydrogen-bonding repeating unit refers to the random selection of a central molecule and the extraction of all the molecules that were hydrogen-bonded to it to form a multi-molecular hydrogen-bonding unit, with the aim of preserving the molecular spatial structure and hydrogen-bonding information as much as possible. We obtained the central molecule by taking the central molecule after optimizing the multi-molecular repeating unit with the GGA/PBE functional [19], a widely used and extensively tested method with proven accuracy in various applications [42,43,44]. The vibrational frequencies were then calculated to investigate the influence of proximity to the original material structure. Additionally, for the multi-molecular fragment model, we further processed the optimized multi-molecular repeating unit by removing structures farther away from the central molecule and only preserving the portion connected to it through hydrogen bonds or conjugated structures, thus, saving computational resources. The construction process of the three models is depicted in Figure 9.

The structural optimization of the single-molecular and minimum repeating units was performed at the GGA/PBE functional level under vacuum conditions. PBE is the recommended default exchange–correlation functional, especially for studies of molecules interacting with metal surfaces, but also widely-used and reliable for bulk calculations [19]. Convergence criteria were specified independently for maximum energy change, maximum force and maximum displacement, these being 1 × 10^−5^ Hartree, 0.002 Hartree Å^−1^ and 0.005 Å, respectively. The vibrational frequencies of the single-molecular and multi-molecular fragment were simulated at the same level of theory as the geometrical optimizations, and the MAEs between the calculated and experimented frequencies were compared to validate the accuracy of the new method. All calculations were conducted by the DMol3 module in Material Studio 2017 program package [45], utilizing a Dell Precision 7670 Workstation.

## 4. Conclusions

In summary, our study presents a novel methodology for simulating equilibrium geometry harmonic vibrational frequencies using multi-molecular fragment interception models. The results showed that the traditional single-molecular simulation and scaling method deviated significantly from the experimental results, while the central-molecular model was better but still not accurate enough, especially for hydrogen-bonded functional groups. Moreover, using the central-molecular model for vibrational frequency calculation resulted in unstable computational accuracy for different drug molecules. The multi-molecular fragment model, on the other hand, demonstrated the highest calculation accuracy with minimum simulation deviation, by preserving the configuration information and intermolecular interactions. The study also provided complete vibrational frequencies calculations and assignments for the three pharmaceutics, which could provide more accurate interpretations of infrared spectra and have never been thoroughly investigated in previous research. Overall, the proposed method has the potential to subvert the current frequency simulation method and improve the accuracy of vibrational spectrum interpretation.

Despite the potential applications of the method, there are limitations and challenges that must be overcome to further enhance its accuracy and applicability. One limitation is the computational cost of the multi-molecular fragment interception model. The model requires a large number of calculations to account for intermolecular interactions, which can increase computational time and limit its applicability to larger systems. To overcome this limitation, one possible improvement would be to incorporate machine learning approaches to optimize the multi-molecular fragment models and improve computational efficiency. For example, artificial neural networks could be trained to estimate intermolecular potentials, reducing the number of calculations required to accurately describe intermolecular interactions. Another limitation is the scalability of the methodology to larger molecular systems. The multi-molecular fragment interception model is more accurate than traditional single-molecular simulations and scaling methods but has limitations in accurately capturing intermolecular interactions in large systems. To overcome this limitation, one possible future direction would be to explore the use of hybrid functionals, which combine the strengths of different exchange–correlation functionals, to improve the accuracy of vibrational frequency calculations for complex molecular systems. By incorporating these hybrid functionals, we may be able to accurately describe intermolecular interactions in larger systems, expanding the applicability of the method to a wider range of molecular systems.

## Figures and Tables

**Table 1:** A portion of observed and calculated vibrational frequencies with their respective dominant normal modes for FIN, LAM, and REP ^a^.

Name	Assignment	Exp	Single Molecular	Single Molecular after Scaled by Scale Factor
FIN	*ν*N_2_H_35_	3429	3517.12	3481.95
	A:*ν*N_1_H	3349	3514.26	3479.12
	*ν*C_13_H_14_	2914	2924.11	2894.87
	*ν*C_3_=0_1_; *ν*C_1_=C_2_; *β*N_1_H_36_;	1688	1695.16	1678.21
	*ν*C_19_=0_2_; *β*N_2_H_35_;	1668	1693.63	1676.69
	*β*C_8,16_H_9,19_;	1277	1268.44	1255.76
	*ρ*C_14–15_H_15–18_; *γ*N_2_C_19_	766	766.83	759.16
	MAE (for all data) ^b^		12.99	16.99
	RMSE (for all data) ^c^		29.80	27.14
LAM	*ν_as_*N_3_H_3–4_	3383	3656.99	3620.42
	*ν_s_*N_3_H_3–4_	3328	3518.43	3483.25
	*ν*C_1_O_1_	1651	1701.03	1684.02
	*ν*C_3_N_2_; *β*N_3_H_3_	1498	1531.51	1516.19
	*γ*C_2_H_1_; *ρ*C_8_H_9–10_	1030	1041.08	1033.67
	*ρ*N_3_H_3–4_; *β*C_5_H_5_; *γ*C_4,6_H_2,6_; *γ*C_8_H_9–10_; ring prckering vibration;	538	533.74	528.40
	MAE (for all data) ^b^		52.17	50.56
	RMSE (for all data) ^c^		109.50	101.75
REP	*ν*O_4_H_36_	3428	3640.80	3566.16
	*ν*N_2_H_35_	3307	3551.09	3478.29
	*ν*C_12,16_H	2804	2863.56	2804.86
	*ν*C_25_O_3_; *β*O_4_H_36_	1686	1747.65	1711.82
	*ν*C_17_O_1_; *β*N_2_H_35_; *γ*C_18_H_8_	1635	1699.94	1665.09
	*ν*C_7,18_H;N_2_H	1300	1299.66	1273.02
	*γ*N_2_H_35_; *ω*C_18_H_8–9_; *γ*C_7–11_H	474	475.74	465.99
	MAE (for all data) ^b^		26.48	29.39
	RMSE (for all data) ^c^		57.51	44.34

^a^ Frequencies are in cm^−1^; ^b^ MAE in cm^−1^; ^c^ RMSE in cm^−1^; *ν*: stretching; *β*: in-plane bending; *γ*: out-of-plane bending; *ρ:* in-plane rocking.

**Table 2:** Observed and calculated vibrational frequencies and their dominant normal modes for FIN, LAM, and REP ^a^.

Name	Assignment	Exp.	Central Molecular	Single Molecular after Scaled by Scale Factor
FIN	*ν*N_2_H_35_	3429	3522.08	3481.95
	A:*ν*N_1_H	3349	3437.05	3479.12
	*ν*C_13_H_14_	2914	2920.99	2894.87
	*ν*C_3_=0_1_; *ν*C_1_=C_2_; *β*N_1_H_36_;	1688	1664.17	1678.21
	*ν*C_19_=0_2_; *β*N_2_H_35_;	1668	1662.83	1676.69
	*β*C_8,13,16_H_9,14,19_	1225	1224.87	1218.55
	*β*N_1_H_36_; *β*C_6,10_H_6–7,10–11_; *δas*C_17,18_H_20–25_	890	882.29	872.55
	MAE (for all data) ^b^		9.34	16.99
	RMSE (for all data) ^c^		21.81	27.14
LAM	*ν_as_*N_3_H_3–4_	3383	3484.85	3620.42
	*ν_s_*N_3_H_3–4_	3327.9	3331.83	3483.25
	*ν*C_1_O_1_	1615	1671.64	1684.02
	*β*N_3_H_3_; *ν*C_3_N_2_	1498	1467.85	1516.19
	*β*C_5,7_H_5,8_; *ω*C_6_H_6–7_; *γ*C_2_H_1_	1184	1182.63	1151.56
	*ω*C_6_H_6–7_; *ν*C_6_S_1_	752	757.16	750.60
	MAE (for all data) ^b^		20.36	50.56
	RMSE (for all data) ^c^		30.40	101.75
REP	*ν*O_4_H_36_	3428	3162.17	3566.16
	*ν*N_2_H_35_	3307	3424.00	3478.29
	*ν*C_25_O_3_; *β*O_4_H_36_	1686	1666.48	1711.82
	*ν*C_17_O_1_; *β*N_2_H_35_; *γ*C_18_H_8_	1635	1621.51	1665.09
	*ν*C_7,18_H; *ν*N_2_H	1300	1304.87	1273.02
	*γ*N_2_H_35_; *γ*C_2,3,4,5_H_6,7,5,4_; *γ*C_12–16_H; *ν*C_1_N_2_	619	610.53	597.38
	MAE (for all data) ^b^		23.12	29.39
	RMSE (for all data) ^c^		52.21	44.34

^a^ Frequencies are in cm^−1^; ^b^ MAE in cm^−1^; ^c^ RMSE in cm^−1^; *ν*: stretching; *β*: in-plane bending; *γ*: out-of-plane bending; *δ*: formation; *ω*: out-plane rocking.

**Table 3:** Vibrational frequencies and dominant normal modes of multi-molecular fragment model and central molecular model for FIN, LAM, and REP ^a^.

Name	Assignment	Exp.	Multi-Molecular Fragment Model	Central Molecular
FIN	*ν*N_2_H_35_	3429	3520.79	3522.08
	A:*ν*N_1_H	3349	3399.51	3437.05
	*ν*C_3_=0_1_; *ν*C_1_=C_2_; *β*N_1_H_36_;	1688	1681.01	1664.17
	*ν*C_19_=0_2_; *β*N_2_H_35_;	1668	1665.21	1662.83
	*β*C_8,13,16_H_9,14,19_	1225	1225.5	1224.87
	*γ*N_1_H_36_; *ρ*C_15_H_17–18_; *δ_as_*C_17_H_20–22_	600	589.69	580.71
	MAE (for all data) ^b^		8.21	9.34
	RMSE (for all data) ^c^		18.35	21.81
LAM	*ν_as_*N_3_H_3–4_	3383	3393.37	3484.85
	*ν_s_*N_3_H_3–4_	3327.9	3232.27	3331.83
	*ν*C_1_O_1_	1651	1653.55	1671.64
	*β*N_3_H_3_; *ν*C_3_N_2_	1498	1498.19	1467.85
	*γ*C_2_H_1_; *ρ*C_8_H_9–10_	1030	1033.46	1031.56
	*γ*N_3_H_3_; *γ*C_5_H_5_	752	757.55	757.16
	MAE (for all data) ^b^		15.95	20.36
	RMSE (for all data) ^c^		26.46	30.40
REP	*ν*O_4_H_36_	3428	3415.14	3162.17
	*ν*N_2_H_35_	3307	3395.98	3424.00
	*ν*C_25_O_3_; *β*O_4_H_36_	1686	1689.41	1666.48
	*ν*C_17_O_1_; *β*N_2_H_35_; *γ*C_18_H_8_	1635	1632.85	1621.51
	*β*C_7–12,14_H; *β*N_2_H_35_	1112	1125.59	1117.81
	*γ*N_2_H_35_; *γ*O_4_H_36_; *γ*C_26_H; ring prckering vibration	763	758.78	743.32
	MAE (for all data) ^b^		12.10	23.12
	RMSE (for all data) ^c^		25.82	52.21

^a^ Frequencies are in cm^−1^; ^b^ MAE in cm^−1^; ^c^ RMSE in cm^−1^; *ν*: stretching; *β*: in-plane bending; *γ*: out-of-plane bending; *δ*: formation; *ρ:* in-plane rocking.

**Table 4:** Comparison of calculation times among different models.

Drug	Process	Single Molecule Model (h)	Central Molecule Model (h)	Multi-Molecule Fragment Model (h)
FIN	Configuration optimization	0.27	4.18	4.18
	Frequency calculation	2.51	2.44	4.10
LAM	Configuration optimization	0.10	15.40	15.40
	Frequency calculation	0.44	0.48	3.26
REP	Configuration optimization	0.26	26.21	26.21
	Frequency calculation	2.45	2.42	7.76

## Data Availability

Not applicable.

## Supplementary Materials

The following supporting information can be downloaded at: https://www.mdpi.com/article/10.3390/molecules28124638/s1, Figure S1: The supercell structures of 2 × 1 × 1 FIN supercell (a), 3 × 3 × 1 LAM supercell (b), and (c) 2 × 1 × 3 REP supercell (Hydrogen bonds are illustrated by blue dashed lines); Table S1: Observed and calculated vibrational frequencies of finasteride with different simulation models; Table S2: Observed and calculated vibrational frequencies of lamivudine with different simulation models; Table S3: Observed and calculated vibrational frequencies of repaglinide with different simulation models.
