# Supplementary material for: Harmonic Vibrational Frequency Simulation of Pharmaceutical Molecules via a Novel Multi-Molecular Fragment Interception Method
Source: Molecules. 2023 Jun 8;28(12):4638. doi: 10.3390/molecules28124638 (PMC10304189; doi:10.3390/molecules28124638)
Supplement: Supplementary file 1 [file molecules-28-04638-s001.zip › molecules-2426606-supplementary.pdf]

Supporting Information

Harmonic Vibrational Frequency Simulation of Pharmaceutical Molecules via a Novel Multi-Molecular Fragment Interception Method

Linjie Wang [1], Pengtu Zhang [1], Yali Geng [1], Zaisheng Zhu [1] and Shiling Yuan [1,2,*]

[1] School of Chemical Engineering, Shandong Institute of Petroleum and Chemical Technology, Dongying 257061, China; linjiewang1989@hotmail.com (L.W.); gogogoooliver@163.com (P.Z.); yaligeng0313@163.com (Y.G.); longinces@126.com (Z.Z.)

[2] School of Chemistry and Chemical Engineering, Shandong University, Jinan 250199, China

* Correspondence: shilingyuan@sdu.edu.cn

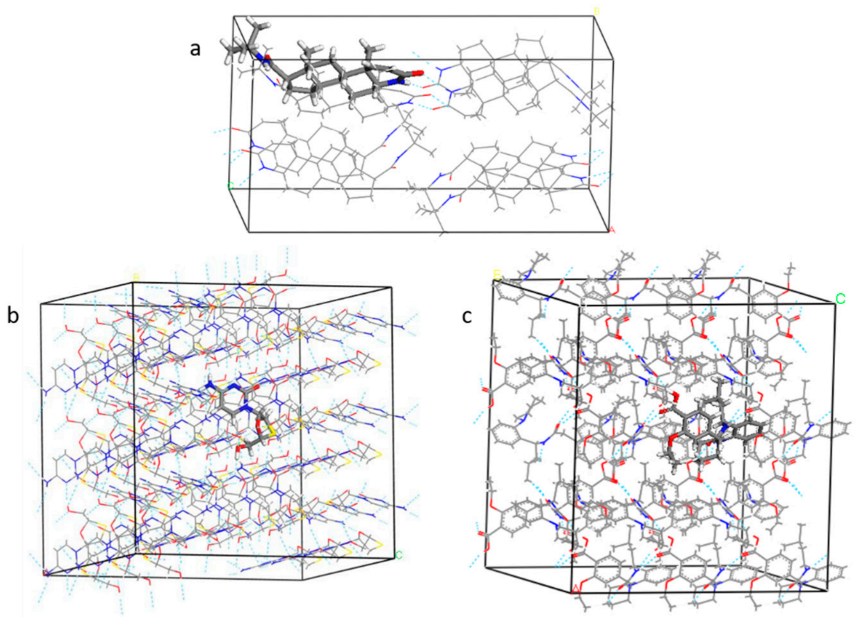

Figure S1. The supercell structures of 2×1×1 FIN supercell (a), 3×3×1 LAM supercell (b), and (c) 2×1×3 REP supercell (Hydrogen bonds are illustrated by blue dashed lines).

Table S1. Observed and calculated vibrational frequencies of finasteride with different simulation models[a].

Assignments	Exp	Single molecular	Simulated single molecular after scaled	Central molecular	Multi-molecular fragment model
νN_2H_{35}	3429	3517.12	3481.95	3522.08	3520.79
A: νN_1H	3349	3514.26	3479.12	3437.05	3399.51
A: νC_2H	3240	3218.12	3185.94	3184.54	3186.75
$\nu_{as}C_{17}H_{20\text{-}22}$	3116	3100.72	3069.71	3115.91	3112.41
$\nu_{s}C_{17}H_{20\text{-}22}$	3036	3022.93	2992.70	3035.73	3034.42
$\nu_{s}C_{6,7}H_{6\text{-}7,8}$	2986	3000.67	2970.66	2988.49	2986.17
$\nu C_{11,16}H_{12\text{-}13,19}$	2969	2973.26	2943.53	2973.68	2975.25
νC_4H_3	2937	/	/	2946.87	2944.37
$\nu C_{13}H_{14}$	2914	2924.11	2894.87	2920.99	2919.15
νC_8H_9	2902	2917.3	2888.13	2905.28	2905.12
νC_4H_3	2866	2885.14	2856.29	N/A	2888.51
$\nu C_3{=}0_1$; $\nu C_1{=}C_2$; βN_1H_{36}	1688	1695.16	1678.21	1664.17	1681.01
$\nu C_{19}{=}0_2$; βN_2H_{35}	1668	1693.63	1676.69	1662.83	1665.21
$\nu C_3{=}0_1$; $\nu C_1{=}C_2$; βN_1H_{36}	1600	1635.8	1619.44	1610.66	1611.10
$\delta_{as}C_{21\text{-}23}H_{26\text{-}34}$	1505	1501.91	1486.89	1502.13	1502.82
$\delta C_{5,6}H_{4\text{-}5,6\text{-}7}$; δ_{as} $C_{17}H_{20\text{-}22}$	1450	1457.6	1443.02	1452.72	1452.24
$\delta_{s}C_{21\text{-}23}H_{26\text{-}34}$	1392	1391.59	1377.67	1392.53	1392.12
$\delta_{s}C_{18}H_{23\text{-}25}$	1383	1382.59	1368.76	1385.39	1385.02
$\delta_{s}C_{21,23}H_{26\text{-}28,32\text{-}34}$	1367	1364.04	1350.40	1366.63	1367.08
$\omega C_{10}H_{10\text{-}11}$; $\beta C_{13}H_{14}$	1340	1341.62	1328.20	1339.44	1341.14
$\beta C_{5,6,7,13,16}H_{5,6,14,19}$	1330	1330.51	1317.20	1328.39	1330.37
$\beta C_{7,8,16}H_{8,9,19}$	1291	1295.94	1282.98	1294.16	1294.21
$\beta C_{8,16}H_{9,19}$	1277	1268.44	1255.76	1266.90	1267.84
$\omega C_{10,14,15}H_{10\text{-}11,15\text{-}18}$; $\tau C_{5,11}H_{5\text{-}6,12\text{-}13}$	1257	1262.69	1250.06	1258.27	1258.15
$\beta C_{8,13,16}H_{9,14,19}$	1225	1230.86	1218.55	1224.87	1225.50
$\tau C_{5,10,14}H_{4\text{-}5,10\text{-}11,15\text{-}16}$; $\beta C_{8,13,16}H_{9,14,19}$	1203	1206.75	1194.68	1201.39	1202.21
$\delta C_{16}H_{17}$; βN_2H_{35}	1168	1167.75	1156.07	1163.91	1164.78
$\beta C_{1,2}H_{1,2}$; βN_1H_{36}	1125	1110.81	1099.70	1123.54	1127.09
$\gamma C_{6,13}H_{7,14}$; $\gamma C_{10,11}H_{10\text{-}13}$	1065	1055.08	1044.53	1055.82	1056.48
$\gamma C_{4,7,8,16}H_{3,8,9,19}$	1027	1028.04	1017.76	1029.20	1029.35
$\omega C_5H_{4\text{-}5}$; γC_6H_6	991	995.97	986.01	990.85	990.67
$\gamma C_{1,2}H_{1,2}$	968	978.29	968.51	963.89	964.07
$\gamma C_{1,2}H_{1,2}$; $\gamma C_{16}H_{19}$	960	965.38	955.73	962.20	962.53
$\gamma C_5H_{4\text{-}5}$	937	938.4	929.02	936.98	937.14
$\delta_{as}C_{21\text{-}23}H_{26\text{-}34}$	914	914.04	904.90	914.46	914.75
βN_1H_{36}; $\beta C_{6,10}H_{6\text{-}7,10\text{-}11}$; $\delta_{as}C_{17,18}H_{20\text{-}25}$	890	881.36	872.55	882.27	883.65
$\rho C_{14\text{-}15}H_{15\text{-}18}$; γN_2C_{19}	766	766.83	759.16	761.96	752.94
$\nu C_{20}C_{21\text{-}23}$; $\nu C_{20}\text{-}N_2$	741	732.96	725.63	735.67	731.29
γC_2H_2	688	695.34	688.39	676.40	674.92
γN_1H_{36}; $\rho C_{15}H_{17\text{-}18}$; $\delta_{as}C_{17}H_{20\text{-}22}$	600	598.19	592.21	580.71	589.69

ρC_5H_{4-5}	572	570.03	564.33	561.24	568.64
$\rho C_{14}H_{15-16}$	536	542.55	537.12	536.37	538.57
βC_1H_1; $\gamma C_{2,7}H_{2,8}$; βC_3O_1	502	496.65	491.68	503.06	503.96
$\beta C_{1,2}H_{1,2}$; γN_2H_{35}; $\rho C_{10}H_{10-11}$	484	488.53	483.64	485.44	485.29
$\delta_{as} C_{21-23}H_{26-34}$	463	470.9	466.19	459.73	460.17
γC_1H_1; Deform of ring C	432	428.79	424.50	431.98	432.86
MAE[b]		12.99	16.99	9.34	8.21
RMSE[c]		29.80	27.14	21.81	18.35

[a] Frequencies are in cm^{-1}; [b] MAE in cm^{-1}; [c] RMSE in cm^{-1}; ν: stretching; β: in-plane bending; γ:out-of-plane bending; τ: torsion; δ: formation; ρ: in-plane rocking; ω: out-plane rocking.

Table S2. Observed and calculated vibrational frequencies of lamivudine with different simulation models[a].

Assignments	Exp	Single molecular	Simulated single molecular after scaled	Central molecular	Multi-molecular fragment model
$\nu_{as}N_3H_{3-4}$	3383	3656.99	3620.42	3484.85	3393.37
$\nu_s N_3H_{3-4}$	3327.9	3518.43	3483.25	3331.83	3232.27
νC_7H_8	3271.6	3102.50	3071.48	3217.71	3198.22
νO_3H_{11}	3207	3733.16	3695.83	3175.46	3173.93
νC_4H_2; $\nu_{as}C_6H_{6-7}$	3074	3099.74	3068.74	3041.01	3048.32
$\nu_s C_6H_{6-7}$	2959	3004.31	2974.27	2970.89	2969.29
$\nu_s C_8H_{9-10}$; νO_3H_{11}	2838	2970.68	2940.97	2923.49	2911.61
νC_1O_1	1651	1701.03	1684.02	1671.64	1653.55
νC_1O_1; δN_3H_{3-4}	1611	1637.40	1621.03	1653.38<br>1605.49	1624.64
δN_3H_{3-4}; βO_3H_{11}, $\nu C{=}C$	1572.6	1592.41	1576.49	1516	1527.38
βN_3H_3; νC_3N_2	1498	1531.51	1516.19	1467.85	1498.19
δC_6H_{6-7}	1455	1446.75	1432.28	1443.56	1447.19
γC_4H_2	1398	1382.46	1368.64	1393.15	1399.52
γC_4H_2; ωC_8H_{9-10}	1362	1368.28	1354.60	1358.50	1362.55
ωC_8H_{9-10}	1340	1322.16	1308.94	1346.78	1343.33
$\gamma C_{2,8}H_{1,10}$	1316	1303.13	1290.10	1311.89	1316.13
βN_3H_3; γC_4H_2; βC_7H_8; νC_1N_2	1287	1268.17	1255.49	1277.09	1282.17
βC_7H_8; τC_8H_{9-10}; $\gamma C_{2,4}H_{1,2}$	1259	N/A	N/A	N/A	1251.35
βC_7H_8; ωC_6H_{6-7}; τC_8H_{9-10}; $\gamma C_{2,4}H_{1,2}$;	1240	1228.43	1216.15	1277.09	1238.69
$\gamma C_{2,4}H_{1,2}$; τC_8H_{9-10}; βO_3H_{11}; ωC_6H_{6-7}; βC_7H_8	1223	1225.43	1213.18	1223.85	1235.09
$\beta C_{5,7}H_{5,8}$; ωC_6H_{6-7}<br>γC_2H_1	1184	1163.19	1151.56	1182.63	1188.06
$\gamma C_{2,6}H_{1,6}$; βC_5H_5	1162	1149.13	1137.64	1162.02	1167.67
ρN_3H_{3-4}<br>D: ρCH_2; $\delta_{as}CH_3$; βOH	1123	1063.18	1052.55	1104.76	1126.33
$\beta C_{5,7}H_{5,8}$; $\gamma C_{4,8}H_{2,9}$; τC6H6-7; B: ρNH_2	1091	1061.1	1050.49	1069.16	1073.97
γC_8H_{9-10}; βC_5H_5	1059	N/A	N/A	N/A	1070.52
γC_2H_1; ρC_8H_{9-10}	1030	1041.08	1030.67	1031.56	1033.46
ρN_3H_{3-4}; $\beta C_{5,7}H_{5,8}$; τC_6H_{6-7}	988	983.53	973.69	971.66	987.81
$\gamma C_{5,7}H_{5,8}$	920	941.27	931.86	900.84	922.85
$\gamma C_{2,4,6}H_{1,2,6}$; γC_8H_{9-10}; ring prckering vibration; γO_3H_{11}	852	829.9	821.60	825.70	835.01
$\gamma C_{2,4}H_{1,2}$; ρC_6H_{6-7}; ring prckering vibration	809	791.19	783.28	811.38	815.89
$\gamma C_{5,7}H_{5,8}$; γN_3H_3	791	758.18	750.60	775.27	783.20
γN_3H_3; γC_5H_5	752	753.15	745.62	757.16	757.55
ωC_6H_{6-7}; νC_6S_1	723	726.28	719.02	735.62	740.62
ωN_3H_{3-4}	670	N/A	N/A	655.85	673.34

$\gamma N_3H_{3\text{-}4}$; $\beta C_{5,7}H_{5,8}$; $\rho C_6H_{6\text{-}7}$; ring prckering vibration;	645	626.28	620.02	628.51	635.83
γC_4H_2; $\gamma C_6H_{6\text{-}7}$; $\gamma C_8H_{9\text{-}10}$; ring prckering vibration	627	608.98	602.89	602.5	609.44
$\rho C_6H_{6\text{-}7}$; βN_3H_3; ring prckering vibration	595	574.54	568.79	575.76	593.87
$\rho N_3H_{3\text{-}4}$; βC_5H_5; $\gamma C_{4,6}H_{2,6}$; $\gamma C_8H_{9\text{-}10}$; ring prckering vibration;	538	533.74	528.40	521.67	525.56
$\rho C_6H_{6\text{-}7}$; $\gamma C_8H_{9\text{-}10}$	460	506.34	501.28	500.63	504.48
$\rho C_8H_{9\text{-}10}$	442	454.33	449.79	432.92	444.70
$\rho N_3H_{3\text{-}4}$	406	444.26	439.82	N/A	427.47<br>415.31
MAE[b]		52.17	50.56	20.36	15.95
RMSE[c]		109.50	101.75	30.40	26.46

[a] Frequencies are in cm^{-1}; [b] MAE in cm^{-1}; [c] RMSE in cm^{-1}; ν: stretching; β: in-plane bending; γ:out-of-plane bending; τ: torsion; δ: formation; ρ: in-plane rocking; ω: out-plane rocking.

Table S3. Observed and calculated vibrational frequencies of repaglinide with different simulation models[a].

Assignments	Exp	Single molecular	Simulated single molecular after scaled	Central molecular	Multi-molecular fragment model
νO_4H_{36}	3428	3640.80	3566.16	3162.17	3415.14
νN_2H_{35}	3307	3551.09	3478.29	3424.00	3395.98
$\nu_{as}C_{26}H_{12-13}$; $\nu C_{27}H_{28-29}$	3100	3088.11	3024.80	3097.45	3098.96
$\nu C_{10}H_{25-27}$	3067	3071.75	3008.78	3071.76	3071.46
$\nu_s C_{13}H_{18-19}$	3029	3011.58	2949.84	3023.50	3022.52
$\nu_s C_{14}H_{20-21}$	2934	2985.82	2924.61	2980.48	2980.70
$\nu_{as}C_8H_{10-11}$	2868	2988.77	2927.50	2973.77	2976.41
$\nu C_{16}H_{17}$	2852	2872.63	2813.74	2874.77	2874.11
$\nu C_{12,16}H$	2804	2863.56	2804.86	2867.34	2866.79
$\nu C_{25}O_3$; βO_4H_{36}	1686	1747.65	1711.82	1666.48	1689.41
$\nu C_{17}O1$; βN_2H_{35}; $\gamma C_{18}H_8$	1635	1699.94	1665.09	1621.51	1632.85
$\beta C_{1-6}H$; deformation of Ph	1491	1491.12	1460.55	1491.80	1493.52
$\delta C_{9-11}H$, $\delta C_{13-16}H$	1450	1461.81<br>1454.04	1431.84<br>1424.23	1456.34<br>1453.87	1457.06<br>1455.69
$\tau C_{26,27}H$; $\beta C_{19-24}H$; βO_4H_{36}; deformation of Ph	1430	1420.69	1391.57	1415.36	1421.02
$\delta_{as}C_{10,11}H_{25-27,31-33}$	1384	1393.04	1364.48	1386.51	1385.81
$\tau C_{26}H_{12-13}$; βO_4H_{36}; deformation of Ph	1339	1346.64	1319.03	1368.72	1350.85
$\omega C_{12,14,15}H_{14-15,20-21,22-23}$; $\gamma C_{16}H_{17}$	1340	1339.08	1311.63	1344.94	1343.67
$\gamma C_{7,18}H$; ωN_2H	1300	1299.66	1273.02	1304.87	1297.91
$\tau C_{26}H_{12-13}$	1285	1307.81	1281.00		1286.07
$\gamma C_{7,8,9,26}H_{34,11,24,12}$; βN_2H_{35}; $\beta C_{20,23,24}H_{1,2,3}$; βO_4H_{36}	1255	1277.16	1250.98	1266.85	1266.90
$\gamma C_{12-16}H$; ωC_8H	1217	1213.99	1189.10	1218.40	1218.69
γC_7H_{34}; $\beta C_{2,3,4}H_{6,7,5}$	1175	1175.75	1151.65	1183.85	1183.63
$\gamma C_{18,20,24,26,27}H$	1151	1165.70	1141.80	1142.98	1150.83
$\beta C_{7-12,14}H$; βN_2H_{35}	1112	1115.76	1092.89	1125.59	1117.81
$\beta C_{2,3,4,5}H_{6,7,5,4}$; $\gamma C_{7,8}H$; ring prckering vibration	1091	1090.21	1067.86	1080.20	1080.27
βN_2H_{35}; $\beta C_{7,8}H$	1040	1045.10	1023.68	1039.70	1042.69
βO_4H_{36}	983	1025.67	1004.64		987.29
$\delta_{as}C_{10,11,12}H_{25-27,31-33}$	943	961.99	942.27	945.27	945.56
$\gamma C_{18}H$; $\gamma C_{23}H$	921	921.46	902.57	916.14	921.26
νO_2C_{26}; $\delta_s C_{27}H_{28-30}$; $\beta C_{20}H$	861	865.25	847.51	851.94	863.35
$\gamma C_{20,24}H_{1,3}$; $\rho C_{26}H_{12-13}$; $\delta_{as}C_{27}H_{28-30}$	804	804.04	787.56	792.14	800.82
$\gamma C_{8,9,10}H_{10,24,25-27}$; deformation of Ph	782	782.99	766.94	775.83	777.41
γN_2H_{35}; γO_4H_{36}; $\gamma C_{26}H$; ring prckering vibration	763	757.16	741.64	743.32	758.78
$\gamma C_{20,24}H_{1,3}$	704	695.73	681.47		701.21
γN_2H_{35}; $\gamma C_{18}H_8$	647	637.95	624.87	618.66	621.77
γN_2H_{35}; $\gamma C_{2,3,4,5}H_{6,7,5,4}$; $\gamma C_{12-16}H$; βC_1N_2	619	609.88	597.38	610.53	619.02

$\gamma C_{2,3,4}H_{6,7,5}$; $\gamma C_{8,18,24}H_{11,8\text{-}9,3}$;	575	581.67	569.75	569.68	571.72
$\rho C_{18}H_{8\text{-}9}$; $\gamma C_{20,23}H_{1,2}$; γO_2C_{21}; $\delta_{as}C_{26}H$	537	546.20	535.00	543.37	535.14
$\gamma C_{12,13,14,15,16}H_{15,18,21,23,16}$	496	489.16	479.13	498.53	498.63
γN_2H_{35}; $\omega C_{18}H_{8\text{-}9}$; $\gamma C_{7\text{-}11}H$	474	475.74	465.99	476.51	471.71
$\gamma C_{12,13,14,15,16}H_{15,18,21,23,16}$	455	456.28	446.93	450.19	452.08
MAE[b]		26.48	29.39	23.12	12.10
RMSE[c]		57.51	44.34	52.21	25.82

[a] Frequencies are in cm^{-1}; [b] MAE in cm^{-1}; [c] RMSE in cm^{-1}; ν: stretching; β: in-plane bending; γ:out-of-plane bending; τ: torsion; δ: formation; ρ: in-plane rocking; ω: out-plane rocking.
